# Modeling and Optimization of Microwave Vacuum Drying for Pinelliae Rhizoma: Integrating Drying Kinetics, Artificial Neural Networks, and Quality Preservation

**DOI:** 10.1002/fsn3.70672

**Published:** 2025-08-15

**Authors:** Pan Wang, Xiaoping Yang, Xiaopeng Huang, Guojun Ma, Shidong Zhang, Siqi Wang, Ming Wang, Zewen Zhu, Yanrui Xu, ZePeng Zang, Xu Liu

**Affiliations:** ^1^ College of Mechanical and Electronical Engineering Gansu Agricultural University Lanzhou China; ^2^ Lanzhou Institute of Husbandry and Pharmaceutical Sciences of Chinese Academy of Agricultural Science Lanzhou China; ^3^ College of Plant Protection Gansu Agricultural University Lanzhou China

**Keywords:** artificial neural networks, drying characteristics, microstructure, microwave vacuum drying, *Pinellia ternata* (Thunb.) Breit, Pinelliae Rhizoma

## Abstract

This study investigated the effects of drying temperature and vacuum degree on the drying characteristics, color, rehydration ratio, nucleoside content, and microstructure of Pinelliae Rhizoma (PR) during microwave‐vacuum drying (MVD). The results demonstrated that MVD significantly reduced the drying time and enhanced processing efficiency. Four different mathematical models and artificial neural network (ANN) predictive models are developed for further comparison. Among the models, the Midilli model provided the best fit for all samples (*R* ^2^ = 0.99012, Pearson's *r* = 0.99509). However, the ANN approach has shown a better performance than the Midilli model. MVD resulted in a decrease in L* values, an increase in a* and b* values, and a higher BI index in dried Pinelliae Rhizoma (PR). No significant difference was observed in Δ*E*, indicating that the combined effects of temperature, vacuum degree, pigment stability, and water migration led to a similar degree of color change. Principal component analysis (PCA) and Pearson's correlation analysis revealed that MVD drying at 40°C and −60 kPa were the optimal conditions for efficiently drying Pinelliae Rhizoma (PR). Under this condition, most nucleosides are well preserved with the exception of adenosine. Overall, MVD technology, as demonstrated in this study, is a promising and suitable drying method in terms of processing efficiency for Pinelliae Rhizoma (PR).

## Graphical abstract

The peeled *Pinellia ternata* (Thunb.) Breit tubers were dried with the microwave vacuum drying oven. Then, its drying characteristics, contents of nucleosides, and surface microstructural alterations of dried samples were determined, respectively. Drying parameters were predicted and optimized using four different mathematical models and artificial neural network (ANN). The results indicate that microwave vacuum drying (MVD) technology is a promising and suitable drying method in terms of processing efficiency for Pinelliae Rhizoma.

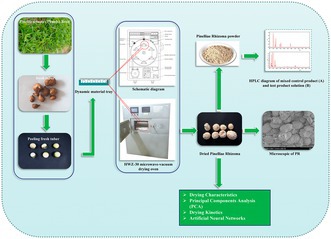

## 1 Introduction

Traditional herbal medicine has been widely used in China due to its potential as a complementary therapy with fewer adverse effects (Zou et al. 2023). Pinelliae Rhizoma (PR), known as Banxia in Chinese, is a well‐established herbal medicine in East Asia derived from the dry tuber of *Pinellia ternata* (Thunb.) Breit. (PT). To date, 233 chemical constituents have been isolated from this herb (Bai et al. 2022). It is extensively utilized in dispensing granules, classical prescriptions, and herbal formulas to treat a wide range of conditions, including cough, infection, phlegm, nausea, asthma, and inflammation (Liang et al. 2025). However, the fresh tuber contains high moisture content, which fosters the growth of pathogenic microorganisms and makes it susceptible to mildew and other degradation reactions, ultimately reducing the levels of active compounds. Drying is a critical step in the post‐harvest management and industrial processing of Pinelliae Rhizoma, which is one of the most common, cost‐effective, and simplest methods for preserving this medicinal material. Currently, China predominantly relies on traditional natural drying methods such as sun‐drying and shade‐drying to dry Chinese herbal medicines (Yang et al. 2024). However, these methods have several disadvantages, including significant quality degradation, long drying time, and low efficiency.

The development of the food industry has provided important support for the drying technologies applied to Chinese herbal medicine. Food industries worldwide have developed different drying techniques to reduce pathogenic bacteria, preserve the nutritional value, minimize agricultural waste, and lower production costs (Al‐Hamdani et al. 2022; Zielinska et al. 2022). Currently, there are several well‐known traditional drying methods such as sun drying, shaded drying, and convective drying. Among these, sun drying is economical but suffers from significant limitations such as dependency on climatic conditions (Pita‐Garcia et al. 2025). Poor weather conditions can lead to issues such as rotting, insect damage, and contamination risks. At present, extensive research has been conducted to explore and develop novel drying technologies such as hybrid solar (Henriques et al. 2025; Pita‐Garcia et al. 2025) and solar‐electric hybrid (Pita‐Garcia et al. 2025), both individually and in combination. Shaded drying is helpful for preserving light‐sensitive substances and minimizing light‐induced chemical reactions such as oxidation (Thamkaew et al. 2021). However, this method has an inevitable limitation, which often requires longer drying times compared with other methods.

Convective drying remains the most used dehydration method in today's traditional herb‐drying processing. However, this method presents several drawbacks, including significant quality degradation, long drying time, and low efficiency, as well as the migration of solutes from the inner parts of the plant to its surface, leading to the phenomenon known as case hardening and uneven shrinkage of the product (El‐Mesery, Qenawy, Li, et al. 2024; Sadeghi et al. 2013). During convective drying, the antioxidant capacity and vitamin C content of orange pomace were observed to decrease with an increase in drying temperature (Afrin et al. 2022). According to (Bai et al. 2023), the different temperatures greatly influenced the color and gingerol content of dried ginger, and the thickness of a ginger slice greatly influenced the rehydration rate using hot air convective drying. Convective drying combined with ultrasound pretreatment technology of cocoyam can reduce processing time, conserve energy, and enhance cocoyam product shelf life (Nkem et al. 2024). Infrared drying has emerged as a promising approach for efficiently drying agricultural products (El‐Mesery, Qenawy, Ali, et al. 2024; Sakare et al. 2020). This drying method involves applying radiation that penetrates moist, porous materials to a certain extent and converts electromagnetic energy into heat (Pawar and Pratape 2017; Boateng et al. 2021), thereby increasing the heat transfer coefficient and energy efficiency (El‐Mesery, ElMesiry, et al. 2025; El‐Mesery, Qenawy, et al. 2025). When integrated with other energy technologies and pretreatment methods, infrared drying can effectively improve food quality while minimizing environmental impact (Abrahão et al. 2024; El‐Mesery, Qenawy, Ali, et al. 2024). In recent years, the microwave drying method has gained popularity due to its ability to uniformly heat plant materials and facilitate rapid water evaporation, resulting in relatively shorter drying times (Zeng et al. 2022). Microwaves will cause the oscillation of water molecules. This oscillation can make the water molecules inside the plant material uniformly absorb microwave energy, resulting in a rapid increase in temperature. In addition, microwaves can cause cell swelling and pore increase in the tissue, which increases the migration rate of water molecules and shortens the drying time (Babaei Rad et al. 2025).

Hybrid drying methods have recently attracted attention for their ability to integrate multiple drying techniques to overcome the limitations of single‐stage processes (El‐Mesery, Qenawy, Ali, et al. 2024). Notable examples include ultrasound contact drying (US‐CD) (Kahraman et al. 2021), contact ultrasonic drying (CUD) (Mundada et al. 2024), convection‐microwave drying (CMD) (Rudy et al. 2024), ultrasound‐assisted microwave vacuum drying (UMVD) (Tu et al. 2025) and microwave vacuum drying (MVD) (Zhu et al. 2024; Ruan et al. 2025). MVD, in particular, has demonstrated promising results by creating large, uniform pores in dried samples, which significantly enhance water evaporation. MVD drying also minimizes adverse effects on product quality and color, shortening the drying time to approximately 38 min, thus presenting a viable option for producing high‐quality dried products (Wang et al. 2025). According to (Zhang et al. 2024), microwave vacuum drying is a superior drying method before extraction that can effectively improve the biological activity of *Rosa roxburghii* Tratt fruit. Accurate prediction of a series of parameters in the drying process, such as drying time, drying temperature, and drying vacuum, is of great significance for reducing energy consumption and stabilizing product quality (El‐Mesery, ElMesiry, et al. 2025; El‐Mesery, Qenawy, et al. 2025).

The ANN method has been widely used to predict and skillfully solve complex nonlinear problems, such as model building, required parameters prediction in the design and development of systems (El‐Mesery, Qenawy, Li, et al. 2024). According to (Liu et al. 2025), the ANN model proved to be a robust tool for predicting and optimizing drying parameters, including drying duration, energy consumption, and quality. Qenawy et al. (2024) used an artificial neural network (ANN) control model to optimize energy consumption, offering a new approach to improving the efficiency of the drying process. Šovljanski et al. (2024) used an artificial neural network (ANN) to optimize untapped potential to enhance different drying methods and their effects on the characteristics of different sweet potato varieties. Therefore, the objectives of the ANN model in this study were to assess the microwave vacuum drying for Pinelliae Rhizoma (PR) and to optimize the drying process.

While MVD has been applied to various herbs, no study has yet combined ANN modeling to predict moisture kinetics and optimize quality retention for Pinelliae Rhizoma. Given the significant medicinal and economic value of Pinelliae Rhizoma, this study aims to: (1) evaluate the effect of MVD on the drying kinetics and quality characteristics of Pinelliae Rhizoma, including color change, microstructure, and functional composition; (2) determine the quality attributes including the nucleosides such as uridine, guanosine, adenosine, inosine, and thymidine content of dried products; (3) study the overall effects of physicochemical qualities after drying by principal component analysis (PCA) and Pearson's correlation analysis. The findings of this study are expected to provide valuable insights for the development of suitable high‐efficiency and uniform heating drying methods for Pinelliae Rhizoma processing.

## 2 Materials and Methods

### 2.1 Materials

Fresh tubers of *Pinellia ternata* (Thunb.) Breit were purchased from a local market in China and stored in a refrigerator at 4°C until further analysis. Prior to drying, the tubers were peeled using a peeling machine (TTP‐35, Tianyang Machinery Company Ltd., Shandong, China) for *Pinellia ternata* (Thunb.) Breit. Afterward, the peeled tubers were immersed in a 4% w/w solution of sodium metabisulfite (Na_2_O_5_S_2_) at 25°C to prevent enzymatic and non‐enzymatic browning, as recommended by (Rasouli et al. 2011).

### 2.2 Experimental Reagents

Guanosine, uridine, adenosine, inosine, uracil, and hypoxanthine, all of which are standard compounds, were purchased from Shanghai Macklin Biochemical Co. Ltd. Acetonitrile (HPLC grade) was obtained from Chengdu Reflex Biotechnology Co. Ltd. (Sichuan, China).

### 2.3 Description of the MVD Dryer

**FIGURE 1:**
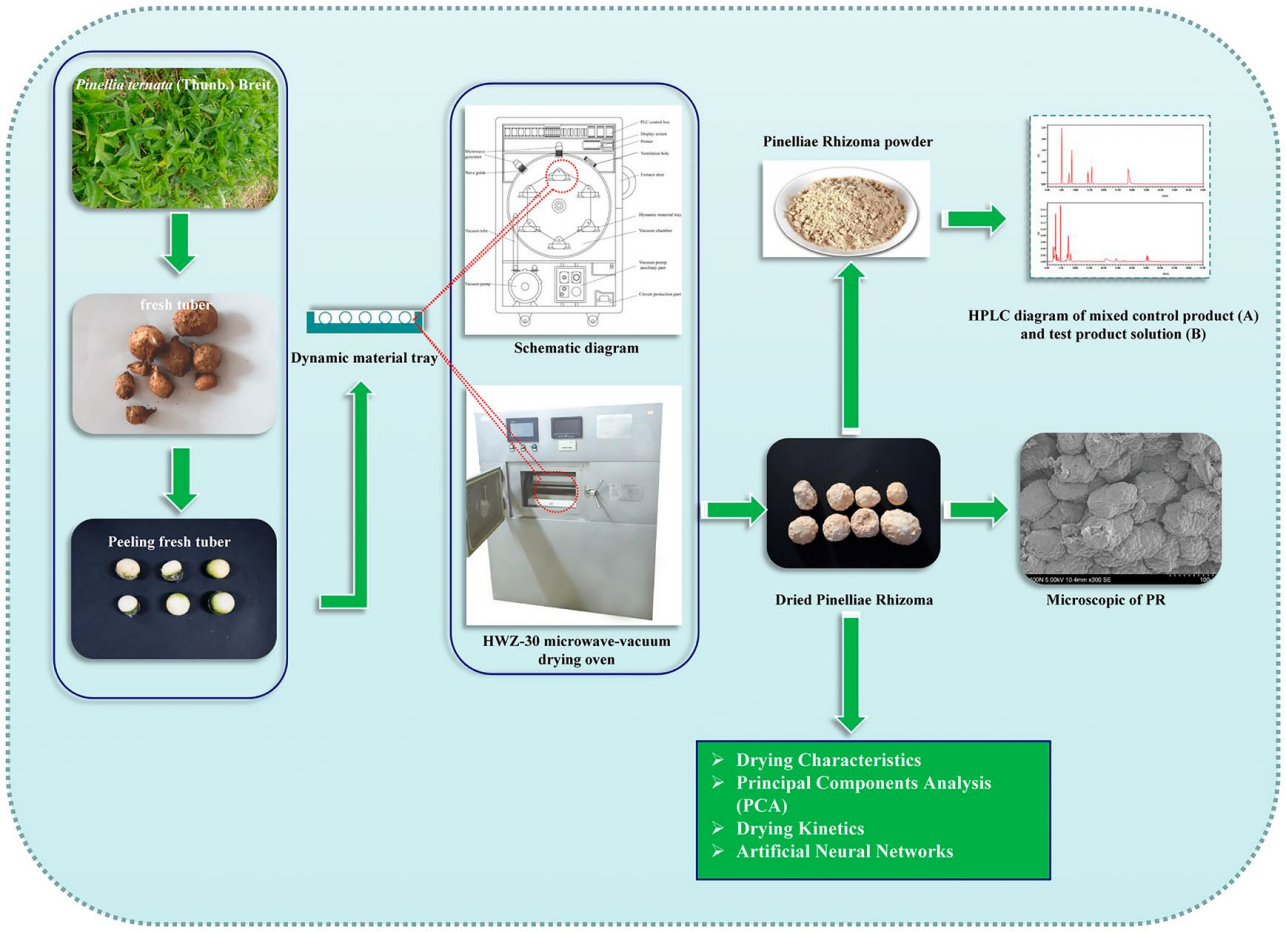
Diagram of the overall research approach.

The peeled tubers drying process was carried out with the microwave‐vacuum drying oven (A HWZ‐30, Tianshui Shenghua Microwave Technology Company Ltd. China). The heating system is composed of two sources which include a microwave generator and a vacuum pump. The samples were spread in a single layer on the feeder tray (400 mm × 150 mm × 100 mm). The microwave power (3 kW) and processing time were adjusted using a digital display. The diagram of the overall research approach is displayed in Figure 1.

### 2.4 Drying Process

The experiment employed various combinations of vacuum degree and temperatures. Specifically, three levels of vacuum degree (−60, −65, and −70 kPa) and temperatures (35°C, 40°C and 45°C) were utilized (Shaw et al. 2007; Zang et al. 2023). The peeled tubers were divided into three groups: natural drying group, hot air drying group, and microwave vacuum drying (MVD). In each experiment, 200 g of peeled tuber samples was positioned in the drying chamber. The samples were weighed continuously using an Electronic Analytical Balance (AX124ZH, OHAUS, Shanghai, China) until their moisture content reached a final moisture content of approximately 10% (wet basis). To ensure the authenticity of the results, each drying condition was repeated three times, and the values were averaged for subsequent research and analysis.

### 2.5 Drying Characteristics

#### 2.5.1 Calculation of Dry Basis Moisture Content

The dry basis moisture content (*M*_*t*_) is calculated as follows (Wang et al. 2023).

(1) $$\;M_{t}=\frac{W_{t}-G}{G}$$

where *W*_*t*_ is the weight of PR at time *t*, and *G* is the absolute mass of dry matter of PR.

#### 2.5.2 Moisture Ratio

The moisture ratio (*M*_*R*_) was calculated using the following formula (Wang et al. 2023).

(2) $$M_{R}=\frac{M_{t}}{M_{0}}$$

Where *M*_*t*_ and *M*_0_ indicate moisture content at time and the initial moisture content, respectively.

#### 2.5.3 Drying Rate

The drying rate (DR) reveals the moisture loss per unit time, which could be calculated using the following formula (Kahraman et al. 2021).

(3) $$\mathrm{DR}=\frac{M_{t1}-M_{t2}}{t_{2}-t_{1}}$$

Where *t*_1_ and *t*_2_ indicate the drying time, min; and *M*_*t*1_ and *M*_*t*2_ denote the dry basis moisture content at time *t*_1_ and *t*_2_, respectively.

#### 2.5.4 Rehydration Ratio

The powder of dried tuber 2.0 g was immersed in the 100 mL distilled water until the weight of the samples reached a constant value. Three replications were performed for each experiment. The rehydration ratio of the dried tuber was calculated using the following formula (Feng et al. 2021).

(4) $$\text{The rehydration ratio}=\frac{D_{2}}{D_{1}}$$

Where *D*_2_ represents the weight of the rehydrated product and *D*_1_ the weight of the dehydrated product, respectively.

#### 2.5.5 Color Measurement

The color measurements of the dried tuber of *Pinellia ternata* (Thunb.) Breit were carried out using a colorimeter (CR‐410, Konica Minolta, Tokyo, Japan). The L*, a*, and b* values displayed on the device screen represent lightness/darkness, redness/greenness, and yellowness/blueness, respectively. Based on the three color parameters, the total color difference (Δ*E*) between the fresh and dried samples was calculated according to the formula (Polat 2024).

(5) $$∆E=\sqrt{{\left(∆L^{*}\right)}^{2}+{\left(∆a^{*}\right)}^{2}+{\left(∆b^{*}\right)}^{2}}$$

where, ΔL*, Δa*, and Δb* indicate the difference of individual L*, a*, and b* color readings from fresh samples, respectively.

### 2.6 Microstructure

Surface microstructural alterations of dried samples subjected to various drying methods were examined using a scanning electron microscope (S‐4800N, Hitachi Corporation, Japan) at 5.0 kV acceleration voltage. The surface microstructure images were observed after gold spray treatment. After this process, the coated samples were placed in the SEM device, and their micrographs were taken (Xiao et al. 2009).

### 2.7 Determination of Nucleoside Using HPLC

A total of 2.0 g dried sample was placed in 50 mL centrifuge tubes and added to 20 mL distilled water. The mixture was vortexed for 30s and subjected to ultrasound‐assisted extraction for 60 min. The mixture was centrifuged after cooling (10,000 rpm, 10 min). After being passed through a 0.22 μm sterile filter membrane, the supernatant was loaded into a liquid‐phase vial. The dry mass content of nucleoside under different pretreatment conditions is carried out with HPLC (Jiang et al. 2022) with slight modifications. The chromatographic conditions were: Agilent Eclipse XDB Plus C18 column (4.6 mm × 250 mm, 5 μm); the mobile phase solutions were 0.5% acetonitrile (A) and water (B); the flow rate was 1 mL/min, and the running time was 60 min; detection wavelength at 265 nm.

### 2.8 Principal Components Analysis (PCA)

The PCA chart is employed to reduce the dimensionality of complex data, with samples exhibiting similar properties positioned close to one another along the numerical axis (Zang et al. 2023). Physicochemical properties were subjected to PCA and Pearson correlation analysis. The scores derived from each PCA were then analyzed using one‐way analysis of variance (ANOVA) to assess significant differences between the samples (Utrilla‐Vázquez et al. 2019).

### 2.9 Drying Kinetics and Artificial Neural Networks

#### 2.9.1 Drying Kinetics

The drying kinetics from the dried samples were matched against 4 distinct thin‐layer models enumerated, including Midilli model, Page model, Henderson and Pabis model, and Two‐Term Exponential model (Table 1). MR, a, k, n, and t within the equations correspond to the moisture ratio, coefficient of the equation, drying constant (min^−1^), exponent, time (min) (Chua et al. 2019a, 2019b).

**TABLE 1:** four thin‐layer model equations.

Model name	Model equation	References
Midilli	MR = aexp(−kt^n^) + bt	Midilli et al. (2002)
Page	MR = exp(−kt^n^)	Murthy and Manohar (2014)
Henderson and Pabis	MR = aexp(−kt)	Demiray and Tulek (2014)
Two‐Term Exponential	MR = aexp(−kt) + (1 − a)exp.(−kat)	Evin (2011)

#### 2.9.2 Artificial Neural Networks

**FIGURE 2:**
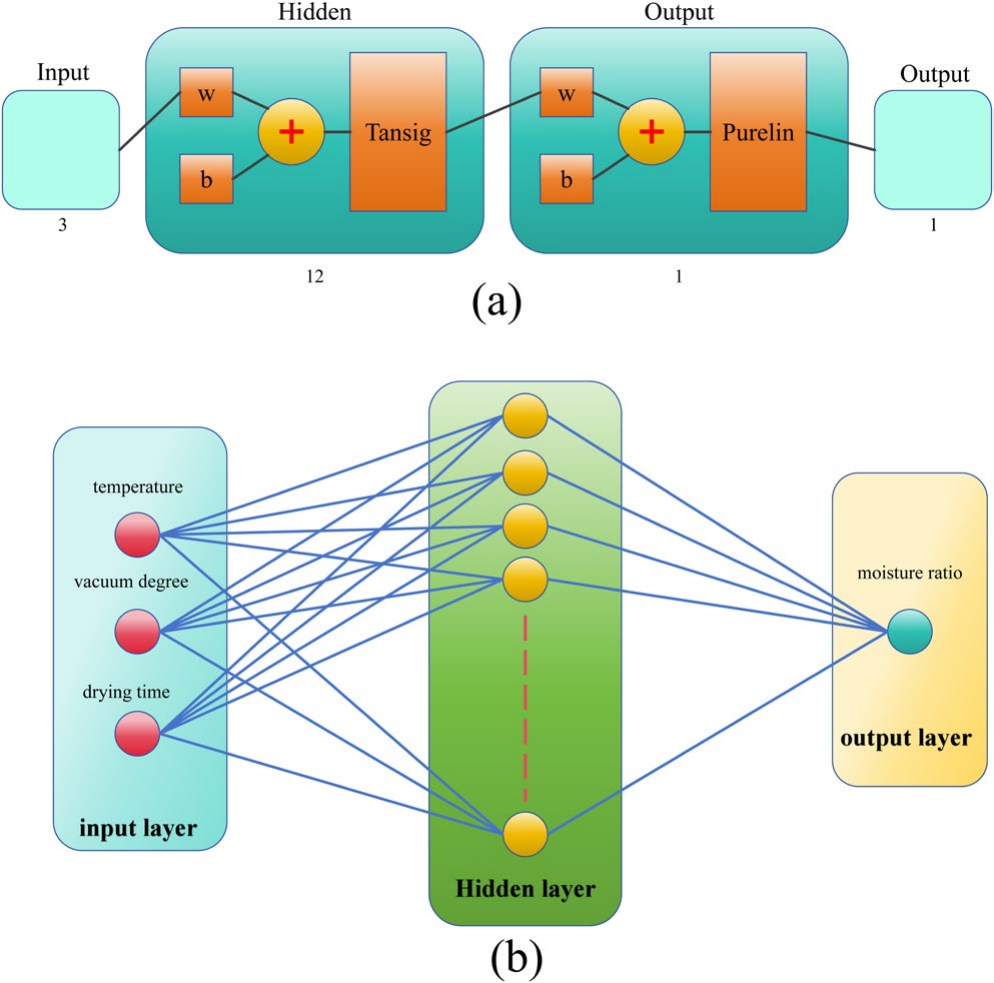
Schematic diagram of ANNs with three input and one hidden layer for moisture ratio: (a) ANN workflow, encompassing both training and prediction stages. (b) ANN architecture.

Artificial neural networks (ANNs) were applied to reproduce the identical experimental data utilized for the Moisture Ratio (MR) during PR drying based on a three‐layer feed forward structure (input layer, hidden layer, output layer), with all processes implemented in MATLAB 2021a (El‐Mesery, Qenawy, Ali, et al. 2024). The workflow of the artificial neural network (including the training and prediction stages) is shown in Figure 2a. The model structure is shown in Figure 2b. The input parameters of the artificial neural network include time, vacuum degree, and temperature, while the predicted output variable is the moisture ratio of PR. This model is trained using the Bayesian Regularization algorithm (trainbr). This algorithm dynamically adjusts the regularization parameters to balance the prediction error and network complexity, which is helpful to prevent overfitting. The setting of the number of layers and nodes in the hidden layer has a significant impact on the performance of the network. Too many will increase the complexity and computational load of the network and even cause overfitting. Too few will affect the performance of the network. Considering the complexity of the network, the hidden layer of the network is generally set to 1 layer, and the number of neurons in the hidden layer is determined according to the empirical formula (Zhao et al. 2021) and the trial‐and‐error method.

(6) $$L=\sqrt{m+n}+a$$

In the formula, where *L* is the number of nodes in the hidden layer, *m* is the number of nodes in the output layer, *n* is the number of nodes in the input layer, and a is the adjustment constant. Training is conducted within the range of 1–10 to find the optimal value. The hidden layer uses the hyperbolic tangent function (tansig) for nonlinear feature extraction, and the output layer uses the linear transfer function (purelin) and applies the physical constraint MR ≥ 0 to ensure that the predicted values conform to the drying kinetic characteristics. The activation function is defined as follows (Serrano et al. 2020):

(7) $$Y_{j}=X_{i}\;\text{purelin}$$

(8) $$Y_{j}=\frac{2}{\left(1+\exp\left(-2X_{j}\right)\right)-1}\;\text{tansig}$$

Where *X*_*j*_ is computed as follows:

(9) $$X_{j}=\sum_{i=1}^{m}W_{\mathrm{ij}}\times Y_{i}+b_{j}$$

where *m* is the number of neurons in the output layer, *W*_*ij*_ is the corresponding weight between *i*th and *j*th layers, *Y*_*i*_ is the *i*th output neuron, *X*_*j*_ is the *j*th input neuron, and *b*_*j*_ is the bias of the *j*th neuron for the related networks. The experimental data set is divided in the proportion of 70% (training set): 15% (validation set): 15% (test set). The validation set is used for real‐time monitoring during the training process. The detailed information of the ANN model is shown in Table 2.

**TABLE 2:** Details of the ANN models.

No	Parameter	Specification
1	Network type	Feed‐Forward Backpropagation (FFBP)
2	Training algorithm	Bayesian Regularization (TRAINBR)
3	Performance function	Mean Square Error (MSE)
4	Activation function	Hidden: TANSIG Output: PURELIN
5	Data division	Fixed ratio (70% train, 15% val, 15% test)
6	Input layer neurons	3 (Time, Temperature, Vacuum)
7	Output layer neurons	1 (Moisture Ratio)
8	Hidden layers	1
9	Hidden neurons	12
10	Maximum epochs	5000
11	Converged at epoch	847
12	Physical constraint	MR ≥ 0

### 2.10 Statistical Analysis

A One‐way analysis of variance (ANOVA) was conducted using SPSS (Version 22.0, IBM SPSS Digital Analytics Co. Ltd., New York, NY, USA) to compare the mean values of the physicochemical properties of *Pinellia ternata* under different groups. Statistical significance was set at *p* < 0.05, and pairwise comparisons were performed using the Least Significant Difference (Fisher's LSD) test.

## 3 Results and Discussion

### 3.1 Effect of Temperature on Drying Characteristics

**FIGURE 3:**
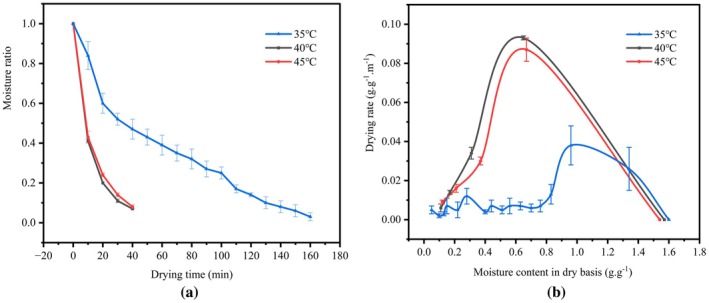
Effect of different drying temperatures on drying characteristics: (a) Moisture ratio curve; (b) drying rate curve.

The effects of drying temperature on the MVD characteristics of PR are shown in Figure 3a. It is evident that drying time significantly decreased as the drying temperature increased from 35°C to 45°C. This can be attributed to the heating of MVD, where higher temperatures facilitated the rapid removal of internal moisture, resulting in a 75% reduction in drying time at both 40°C and 45°C. The drying rate curve indicates that the drying rate of PR increased with the rising drying temperature due to the high amount of available water molecules resulting in high microwave energy absorption (Chua et al. 2019a, 2019b). As shown in Figure 3a, the drying rate at 40°C was higher than at 45°C, possibly due to the surface hardening effect at higher temperatures, which reduced the evaporation rate of water (Zang et al. 2023). Additionally, the drying rate of PR at different temperatures exhibited both an accelerated drying stage and a falling rate drying stage, consistent with previous findings for garlic (Liu et al. 2023). During the initial drying stage, PR samples contain free water between the cells, which is easier to evaporate. In the later drying stages, the amount of free water gradually decreases, and surface hardening inhibits water transfer accordingly.

### 3.2 Effect of Different Vacuum Degree on Drying Characteristics

**FIGURE 4:**
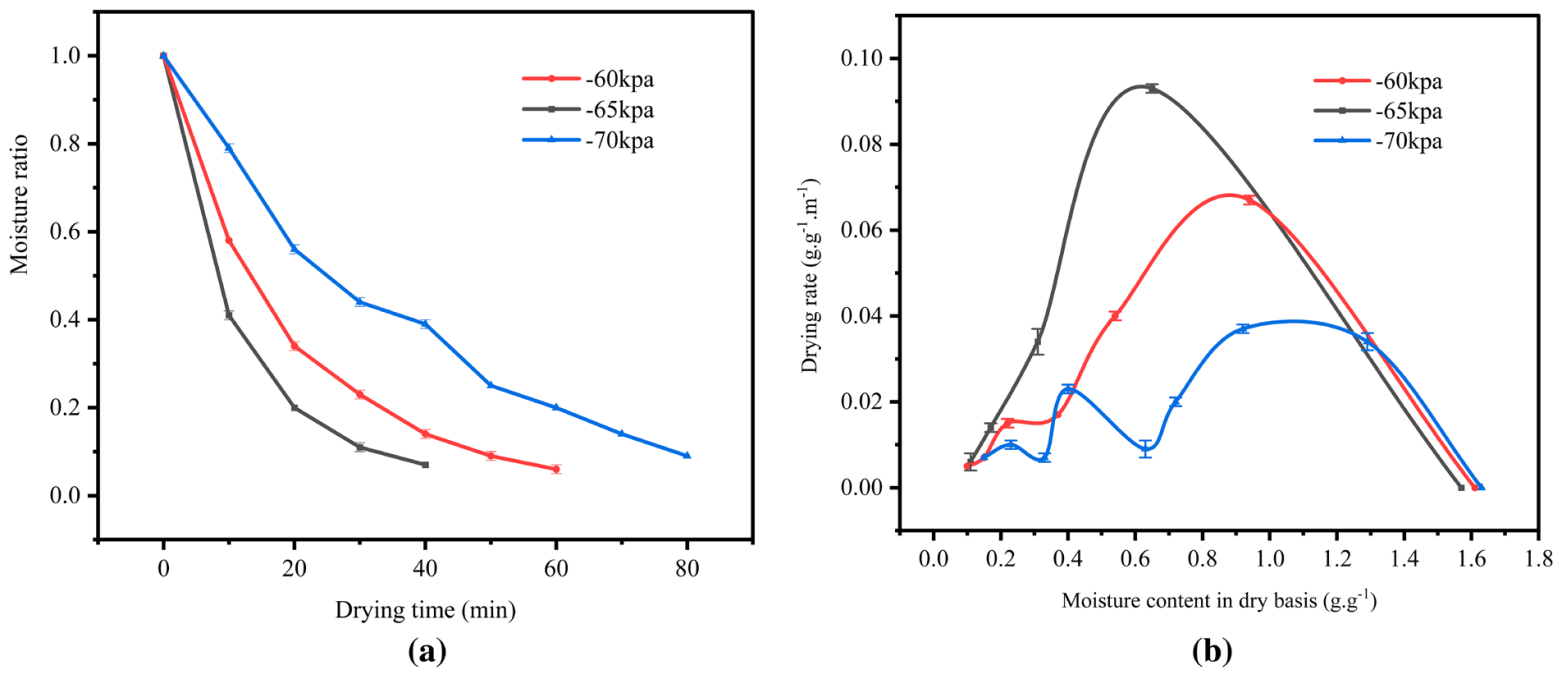
Effect of different vacuum degrees on drying characteristics: (a) Moisture ratio curve; (b) drying rate curve.

The effect of varying vacuum levels on the MVD characteristics of PR is illustrated in Figure 4a. As the vacuum degree decreases (−70, −60, −65 kPa), the drying time of PR reduces (80, 60, and 40 min). This is likely due to the higher vacuum level (−70 kPa), which enhances evaporation efficiency and lowers surface moisture. As drying continues, the decreased surface moisture results in lower microwave absorption, extending the drying time. In contrast, a lower vacuum degree significantly alters the air pressure within the sealed chamber, reducing the saturation vapor pressure of water. This promotes the removal of surface water through external diffusion into the surrounding air under the same heating conditions (Chua et al. 2019a, 2019b). The drying curves in Figure 4b show that the drying rate initially increases and then decreases as moisture content decreases. Notably, at −70 kPa, the drying rate drops, and the drying time increases. Therefore, a higher vacuum degree is more effective in improving drying speed without compromising product quality (Zang et al. 2023).

### 3.3 Rehydration Ratio

**FIGURE 5:**
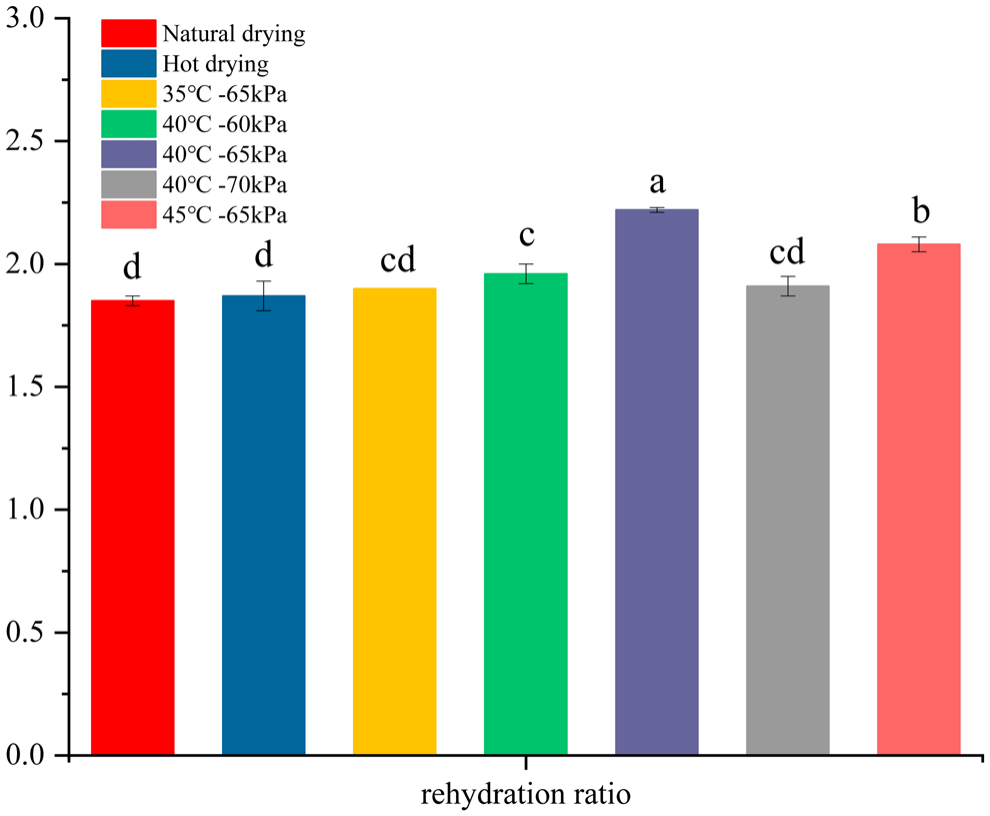
The rehydration ratio of dried PR (*p < 0.05*).

Rehydration is a key quality parameter for dried foods, influenced by both the drying process and the product composition (Salehi et al. 2023). A high rehydration ratio indicates better water absorption and minimal damage to the structure, while a slow or inadequate rehydration ratio suggests internal tissue degradation and collapse (Yi et al. 2015). The experimental data on the rehydration ratio of dried PR are presented in Figure 5. The samples dried by different methods showed significantly different rehydration ratios (*p < 0.05*). The mean rehydration ratios at 35°C, 40°C, and 45°C were 1.90%, 2.22%, and 2.08%, respectively. The treated samples exhibited higher rehydration ratios compared to the control groups (shade drying and hot drying). These results suggest that the dried Pinelliae Rhizoma has a better rehydration capability, likely due to its porous structure, which facilitates moisture diffusion and thus enhances the rehydration ratio.

### 3.4 Color Measurement

**FIGURE 6:**
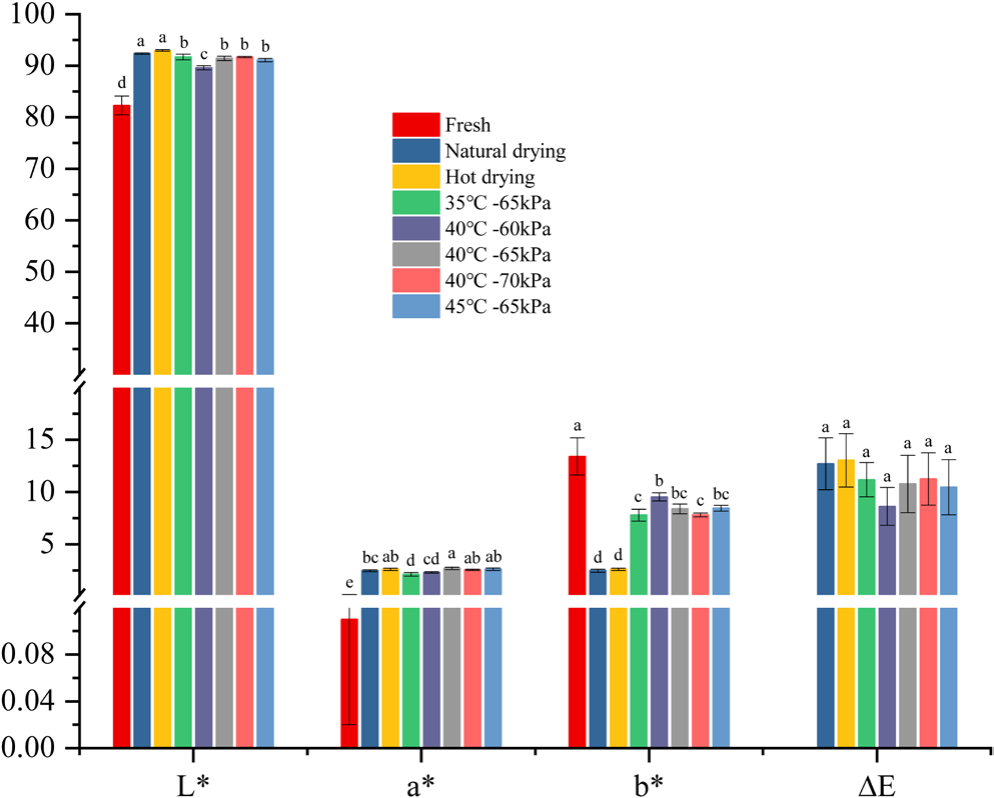
The color parameters and indices of PR at different drying temperatures.

Color is a crucial appearance index of dried Chinese herbal medicine, and undesirable color changes can negatively affect their quality. The surface color parameters of fresh and dried materials under different drying conditions are given in Table 3. MVD treatment could significantly impact the L*, a*, and b* values (*p < 0.05*). Raising the temperature and vacuum degree led to a decrease in L* values, while a* and b* values increased. This could be due to higher temperatures and vacuum pressures promoting interactions between reducing sugars and amino acids in Pinelliae Rhizoma (Chen et al. 2023). As shown in Table 3, the values of the BI increased from 190.61 to 191.48 as the drying temperature increased from 35°C to 45°C, and from 190.25 to 192.72 across different vacuum degrees. This may be attributed to the high vacuum degree reducing oxidation reactions on the surface of PR, while higher temperatures promote the Maillard reaction between polysaccharides and amino acids, significantly increasing the BI (Yang et al. 2022). Conversely, El‐Mesery, ElMesiry, et al. (2025) found that when the temperature was increased from 35°C to 55°C, the BI of dried okra showed a significant reduction of 19.51% and 17.13%, respectively. The reason is that dried Pinelliae Rhizoma is rich in proteins, peptides, amino acids, reducing sugars, and other substances, which cause changes in the color, due to the higher temperatures promoting Maillard reactions. According to (El‐Mesery, Qenawy, et al. 2025), the total color change values (∆*E*) of dried garlic increased with IR and higher air temperature but declined with higher air velocity. Conversely, the total color change values (∆*E*) of dried okra increased in each treatment as the drying temperature increased. This may be due to more severe non‐enzymatic browning at higher temperatures (El‐Mesery, ElMesiry, et al. 2025). However, our findings showed no significant difference compared with natural drying control groups, indicating that the color of MVD drying samples was similar to the natural drying groups (Figure 6). This could be due to the combined effects of temperature, vacuum degree, pigment stability, and water migration, resulting in a similar degree of color change and no significant difference.

**TABLE 3:** Color parameters of raw and dried PR samples.

Parameter	Condition	L*	a*	b*	∆*E*	BI
Fresh	—	82.28 ± 1.83^d^	0.11 ± 0.09^e^	13.41 ± 1.77^a^	0	197.33^a^
Natural drying	—	92.38 ± 0.14^a^	2.47 ± 0.09^bc^	6.12 ± 0.15^d^	12.70 ± 2.48^a^	188.54^e^
Hot drying (°C)	40	92.96 ± 0.16^a^	2.60 ± 0.11^ab^	6.39 ± 0.11^d^	13.04 ± 2.55^a^	188.89^de^
Temperature (°C)	35	91.70 ± 0.56^b^	2.12 ± 0.17^d^	7.79 ± 0.56^c^	11.18 ± 1.64^a^	190.25^cde^
	40	91.44 ± 0.43^b^	2.69 ± 0.12^a^	8.39 ± 0.47^bc^	10.78 ± 2.74^a^	191.42^bc^
	45	91.09 ± 0.33^b^	2.61 ± 0.12^ab^	8.46 ± 0.28^bc^	10.46 ± 2.65^a^	191.48^bc^
Vacuum value (kPa)	−60	89.61 ± 0.39^c^	2.30 ± 0.07^cd^	9.53 ± 0.39^b^	8.62 ± 1.82^a^	192.72^b^
	−65	91.44 ± 0.43^b^	2.69 ± 0.12^a^	8.39 ± 0.47^bc^	10.78 ± 2.74^a^	191.42^bc^
	−70	91.69 ± 0.13^b^	2.56 ± 0.06^ab^	7.81 ± 0.19^c^	11.26 ± 2.50^a^	190.61^cd^

*Note:* The statistics of each color parameter column have been applied separately, and the differences between the means with different letters in the same column are significant (p < 0.05).

### 3.5 Microstructure

**FIGURE 7:**
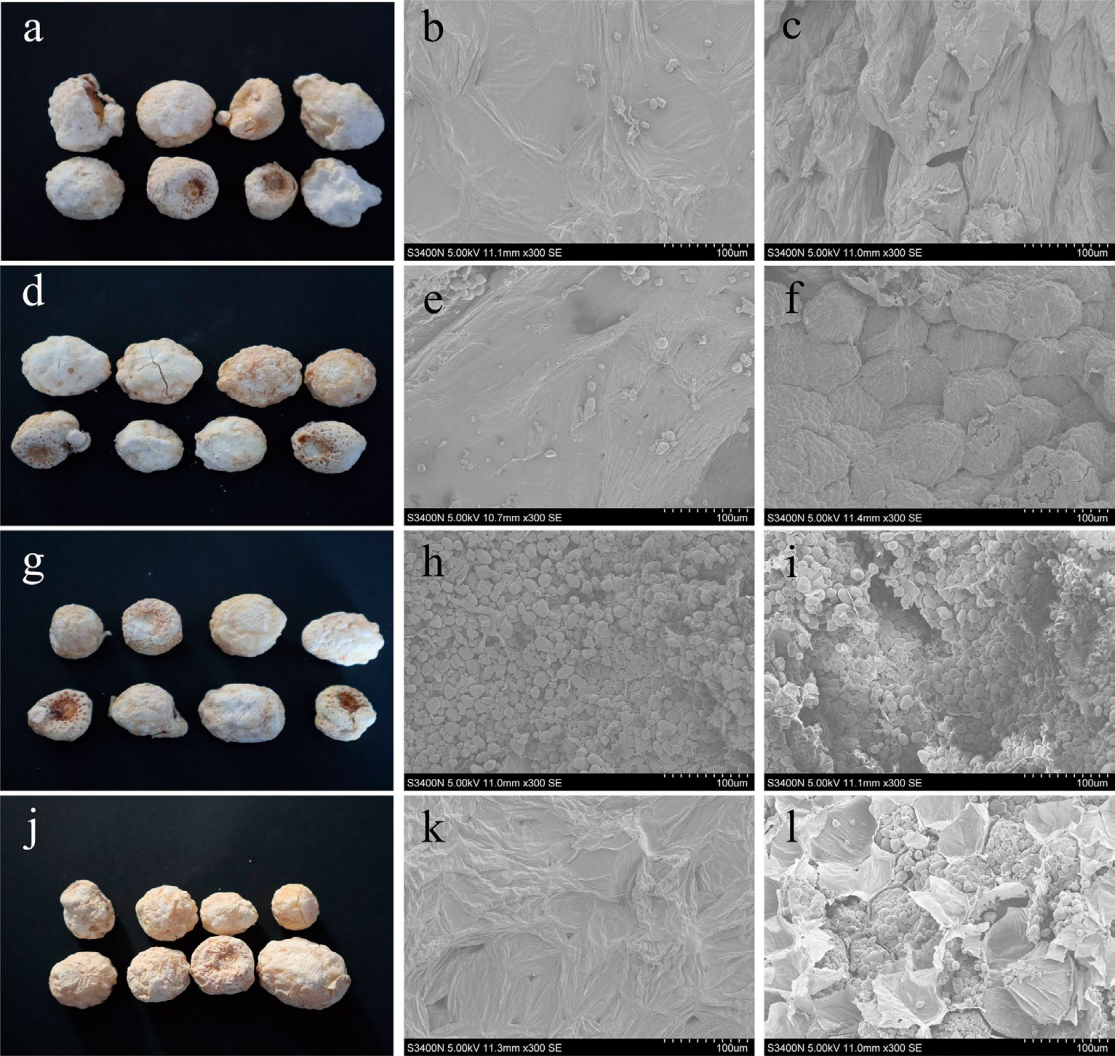
Macroscopic and microscopic images of PR under different drying conditions. (a, b, c) 35°C/−65 kPa, (d, e, f) 40°C/−60 kPa, (g, h, i) 40°C/−70 kPa, (j, k, l) 45°C/−65 kPa. (b, e, h, k) exhibited the surface hardening phenomenon under the different drying conditions, respectively. (c, f, i, l) exhibited the intercellular spaces under the different drying conditions, respectively.

The microstructure of a material plays a crucial role in determining its macroscopic properties. Thermal drying can induce significant structural changes when plant tissues are subjected to simultaneous thermal and moisture gradient stresses. These stresses lead to microstructural deformation and shrinkage as moisture is removed from the cells, resulting in a reduction of turgor pressure (Cichowska‐Bogusz et al. 2020). The scanning electron microscope images of PR dried with MVD are presented in Figure 7. Notable shrinkage of cell volume and partial collapse of the tissue were observed, with the surface microstructure becoming smooth and dense (Figure 7b,c). Furthermore, as the drying temperature increased, the surface microstructure of PR became more compact (Figure 7e), and the internal cell structure also became denser (Figure 7f). This can be attributed to the absorption of a large amount of microwave energy, which causes water vapor to be expelled from the intercellular spaces, leading to an increase in the internal and external vapor pressure gradients. As shown in Figure 7h, a higher vacuum level (40°C/−70 kPa) resulted in substantial microstructural shrinkage, with severe damage to the cell structures. These findings suggest that higher vacuum conditions led to the collapse of cell walls, membrane breakdown, and the formation of larger intercellular spaces, contributing to the deformation of the cell structure. Additionally, as shown in Figure 7h,k, significant surface shrinkage and fracture of the internal cell walls were observed when the drying temperature was increased from 40°C to 45°C. This phenomenon may be due to surface hardening, which generates localized stress, resulting in the disruption of the tissue structure. This observation is consistent with the drying characteristics analysis (Zang et al. 2023).

### 3.6 Drying Kinetics and Artificial Neural Networks

#### 3.6.1 Drying Kinetics

**FIGURE 8:**
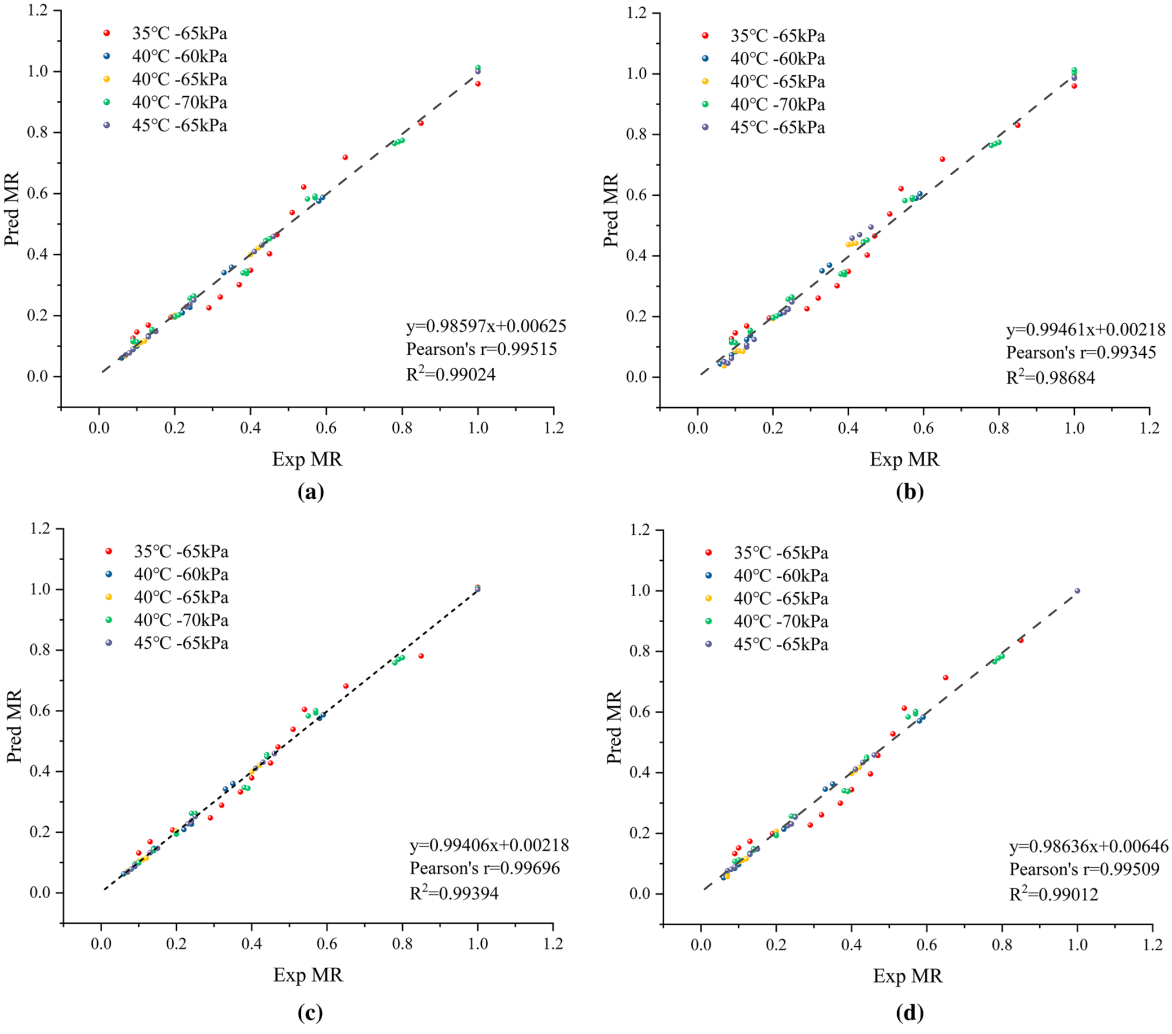
Experimentally determined and predicted moisture ratio by two‐term model (a), Henderson and Pabis model (b), Midilli model (c), and Page model (d) with different drying conditions.

To investigate the drying kinetics of PR, the experimental and predicted moisture contents, derived from regression analysis, are presented in Figure 8. The accuracy of the prediction is indicated by the slope and intercept of the regression curve, with values closer to 1 and 0 representing better prediction accuracy, respectively. As shown in Figure 8, the *R* ^2^ values for the Page model (d), Henderson and Pabis model (b), Two‐Term Exponential model (a), and Midilli model (c) range from 0.98684 to 0.99394, while the Pearson's correlation coefficient values range from 0.99345 to 0.99696. Among these models, the Midilli model demonstrated the best prediction performance. This finding further supports the theoretical understanding of the drying characteristics of PR.

#### 3.6.2 Artificial Neural Networks

**FIGURE 9:**
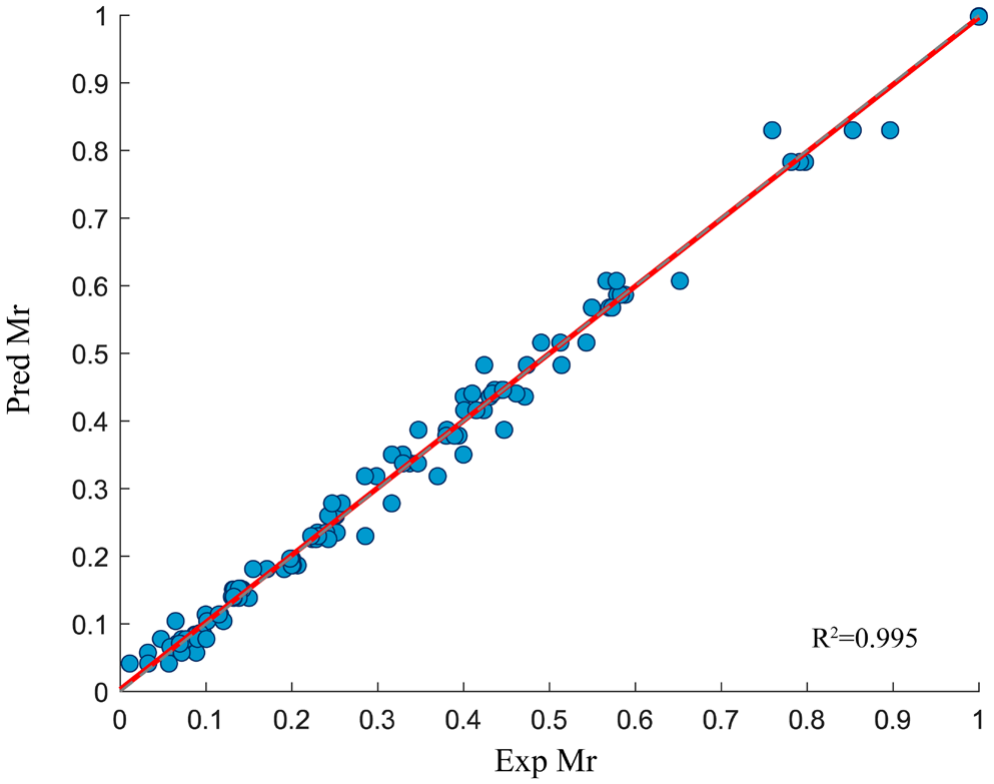
Experimentally determined and predicted moisture ratio by ANN.

**FIGURE 10:**
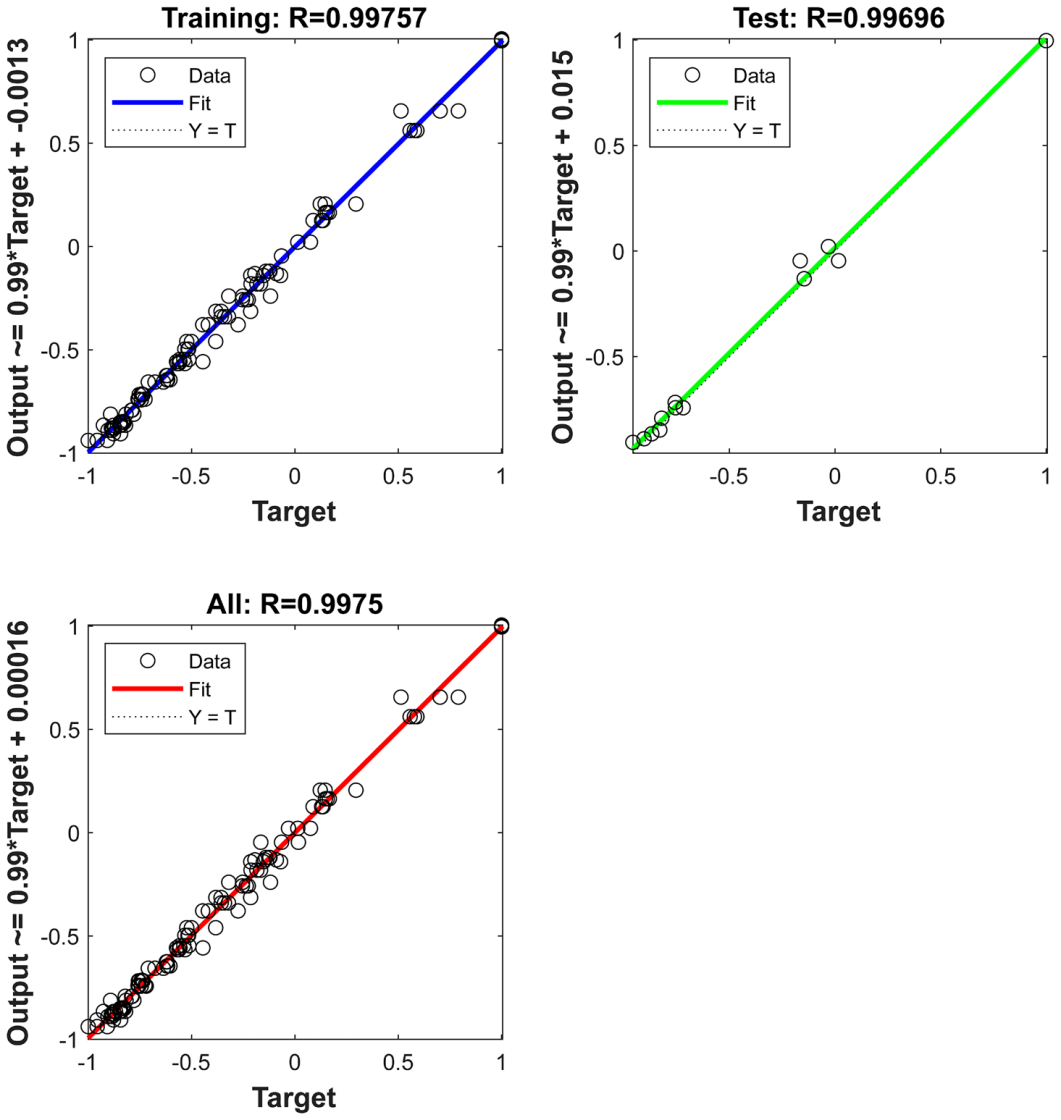
Comparison between the experimental and predicted moisture ratio values during the training and testing of the best ANN model.

In this study, ANN based on the Bayesian Regularization Algorithm (trainbr) was adopted to predict the moisture ratio (MR) during the drying process of *Pinellia ternata* . The hidden layer uses the hyperbolic tangent function (tansig) to enhance nonlinear feature extraction, and the output layer adopts the linear function (purelin) to maintain the continuity of regression (Formulas ((6), (7), (8))). For small sample data (three repetitions per group), the regularization parameters are adaptively adjusted through the Bayesian framework to balance the mean square error (MSE) and network complexity, effectively preventing overfitting. As shown in Figures 9 and 10, the predicted values of ANN are highly consistent with the measured values (*R* ^2^ = 0.995), and the difference between the training set and the test set is small, fully demonstrating that the model still has strong generalization ability in small sample scenarios. The comparative parameters that are calculated based on the data presented in Table 4 show excellent prediction as compared with the experimental data suggested by similar works (Chokphoemphun et al. 2024; Billah et al. 2025).

**TABLE 4:** Comparative study of ANN and the best model (midillli model).

Model	MSE	*R* ^2^
Midilli	0.0055	0.99394
ANN	0.0015	0.995

### 3.7 PCA Analysis and Pearson's Correlation Analysis

**FIGURE 11:**
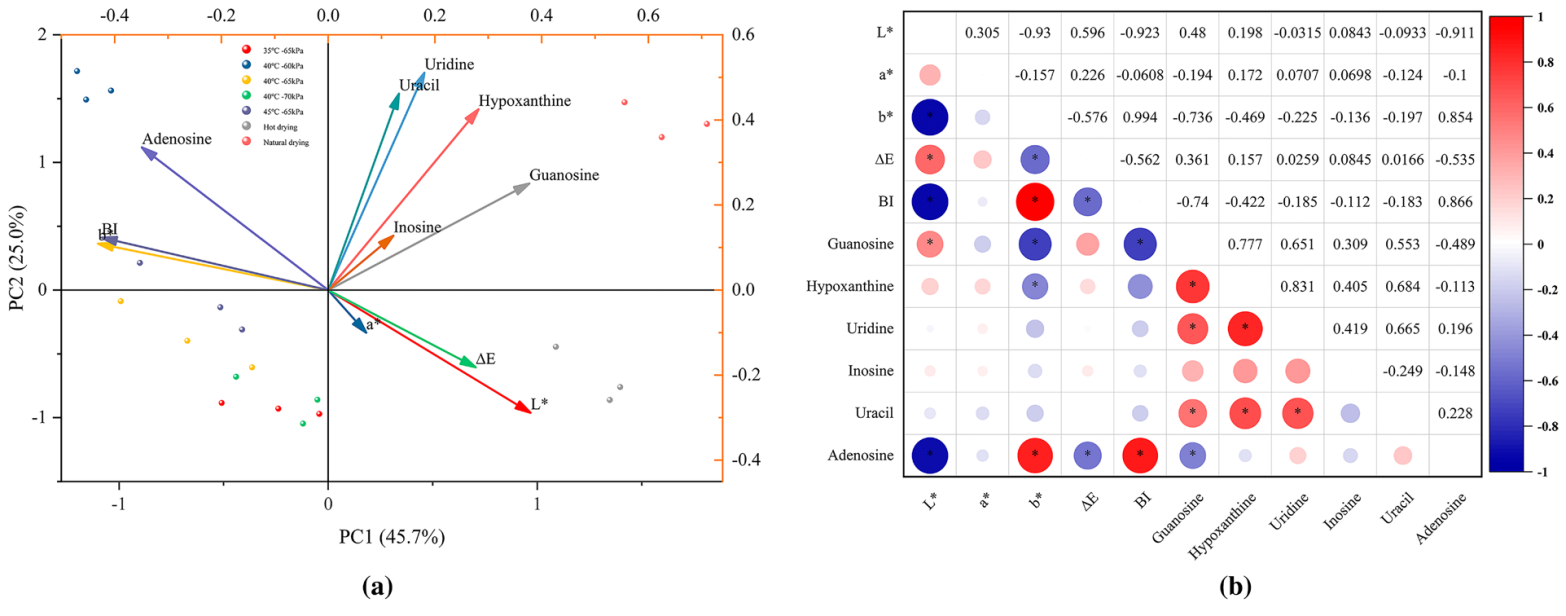
Principal component analysis (a) and Pearson correlation analysis (b) plot of physicochemical quality obtained from different drying treatments.

To comprehensively evaluate the influence of the MVD process on both drying characteristics and the physicochemical properties of the samples, principal component analysis (PCA) was employed to analyze the entire dataset. As illustrated in Figure 11a, the first and second principal components (PC1 and PC2) accounted for 45.70% and 25.0% of the total variance, respectively, thus adequately capturing the inherent variability within the data. Notably, in PC1, a strong positive correlation was observed among guanosine, hypoxanthine, inosine, uracil, and uridine, while adenosine appeared to be dissociated from this core group. The natural and hot drying treatments were positioned on the positive side of PC1, whereas the MVD groups were situated on the negative side. The former exhibited higher concentrations of guanosine, hypoxanthine, and uridine, while the latter were predominantly characterized by elevated adenosine levels, as well as the BI index and b* value. These findings collectively suggest that while MVD (40°C, −60 kPa) significantly harms the surface color of the samples, it has a good impact on the concentration of most nucleosides, with the exception of adenosine. This observation is in alignment with the results of the Pearson correlation analysis (Figure 11b). It can be inferred that, unlike natural and hot drying methods which foster metabolite accumulation, the MVD technique, by reducing drying time, preserves the stability and integrity of bioactive components.

### 3.8 Total Content of Nucleosides

**FIGURE 12:**
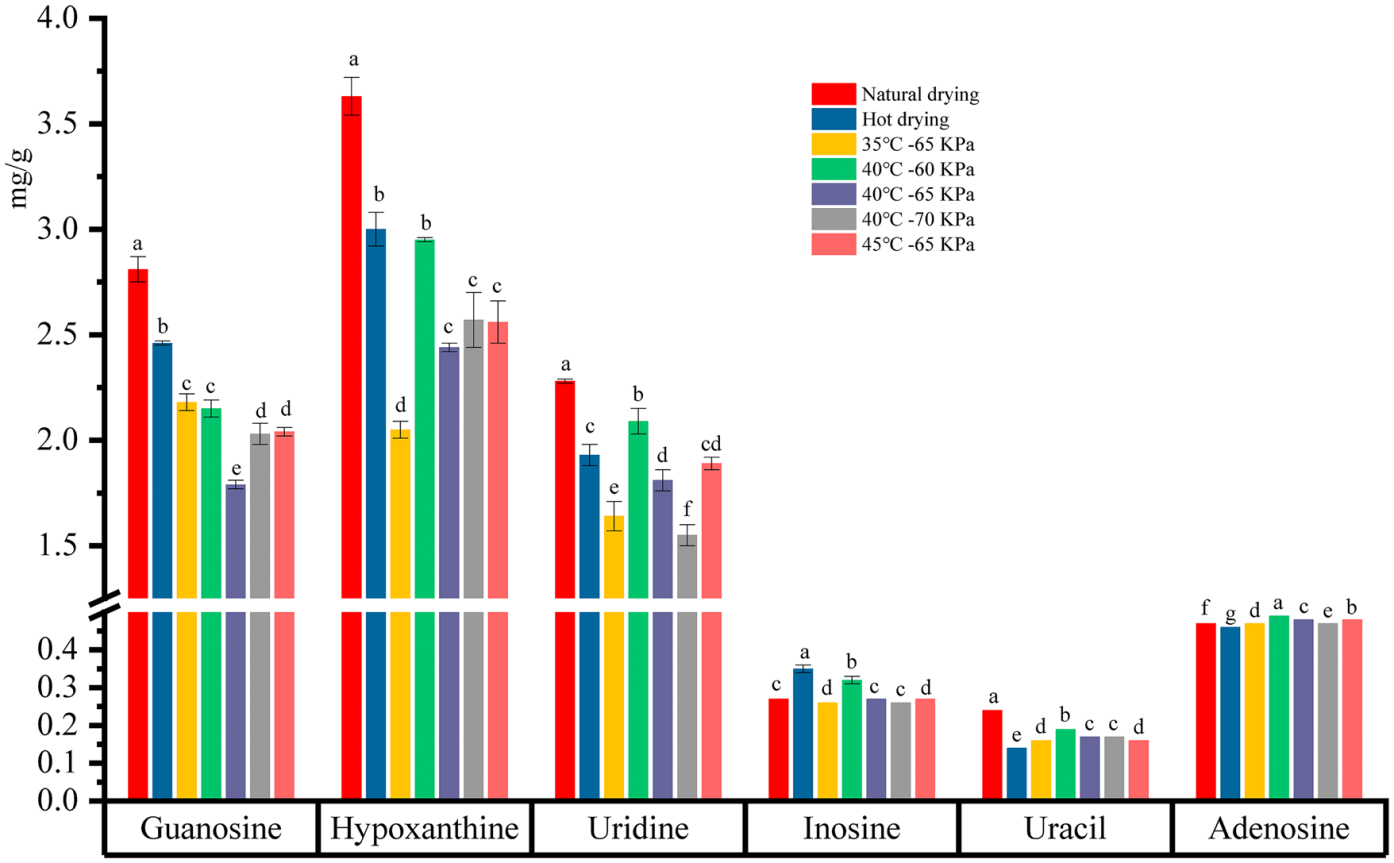
Changes in the content of nucleosides.

**FIGURE 13:**
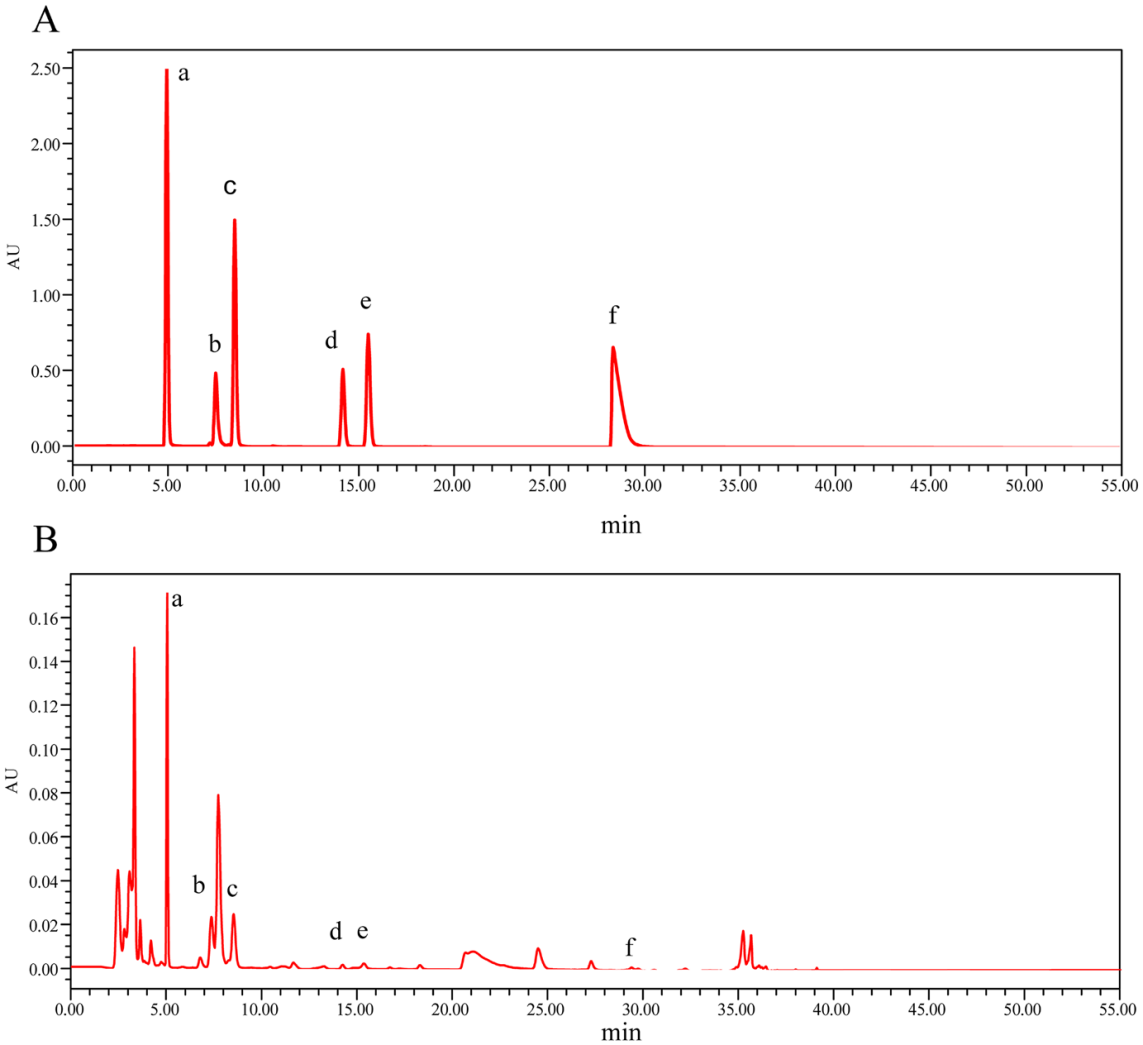
HPLC diagram of mixed control product (A) and test product solution (B). a, Guanosine; b, Hypoxanthine; c, Uridine; d, Inosine; e, Uracil; f, Adenosine.

Currently, the nucleosides such as uridine, guanosine, adenosine, inosine, and thymidine have been identified, which were regarded as characteristic ingredients for evaluating the quality of PR (Qiu et al. 2023). Moreover, the nucleotide signatures have emerged as crucial markers for the authenticity of PR products on the market (Qiu et al. 2023). To explore the influence of microwave vacuum drying methods on the PR appearance characteristics and chemical components, the contents of nucleosides were quantified using the HPLC method (EL‐Mesery and El‐khawaga 2022) (see Figure 13). The chromatogram was recorded and a linear fit was performed on the mass concentrations and peak areas of the mixed controls. The concentration was the horizontal coordinate (mg/g, X) and the peak area (Y) was the vertical coordinate (Figure 12). The results are summarized in Tables 5 and 6.

**TABLE 5:** Results of the examination of the linear relationships of the components (*n* = 6).

Component	Regressive equation	*R* ^2^	Linear range
Guanosine	*y* = 9.7469*x*—973.33	0.97141	130–2130
Hypoxanthine	*y* = 2.8332*x*—82.829	0.99994	140–2170
Uridine	*y* = 7.5655*x*—128.53	0.99981	140–2170
Inosine	*y* = 3.2381*x*—68.845	0.9998	130–2030
Uracil	*y* = 5.1406*x*—67.37	0.9965	120–1930
Adenosine	*y* = 10.537*x*—482.74	0.99947	130–2130

**TABLE 6:** Chromatogram of standard nucleosides analyzed by HPLC.

Parameter	Condition	Guanosine	Hypoxanthine	Uridine	Inosine	Uracil	Adenosine
Natural drying	—	2.810 ± 0.055^a^	3.626 ± 0.094^a^	2.276 ± 0.010^a^	0.275 ± 0.002^c^	0.240 ± 0.003^a^	0.467 ± 0.000^f^
Hot drying (°C)	40	2.455 ± 0.010^b^	3.002 ± 0.077^b^	1.932 ± 0.048^c^	0.346 ± 0.008^a^	0.144 ± 0.001^e^	0.462 ± 0.000^g^
Temperature (°C)	35	2.183 ± 0.042^c^	2.052 ± 0.044^d^	1.636 ± 0.072^e^	0.258 ± 0.001^d^	0.157 ± 0.001^d^	0.473 ± 0.000^d^
	40	1.785 ± 0.017^e^	2.441 ± 0.015^c^	1.808 ± 0.051^d^	0.274 ± 0.000^c^	0.170 ± 0.000^c^	0.475 ± 0.000^c^
	45	2.038 ± 0.018^d^	2.558 ± 0.101^c^	1.886 ± 0.028^cd^	0.268 ± 0.001^c^	0.158 ± 0.001^d^	0.484 ± 0.000^b^
Vacuum degree (kPa)	−60	2.149 ± 0.036^c^	2.949 ± 0.014^b^	2.090 ± 0.056^b^	0.317 ± 0.006^b^	0.188 ± 0.002^b^	0.489 ± 0.001^a^
	−65	1.785 ± 0.017^e^	2.441 ± 0.015^c^	1.808 ± 0.051^d^	0.274 ± 0.000^c^	0.170 ± 0.000^c^	0.475 ± 0.000^c^
	−70	2.030 ± 0.048^d^	2.572 ± 0.134^c^	1.547 ± 0.050^f^	0.256 ± 0.003^d^	0.171 ± 0.003^c^	0.468 ± 0.001^e^

*Note:* Statistical analysis of each nucleoside have been carried out separately, and the difference in mean values represented by different letters in the same column are significant (p < 0.05).

#### 3.8.1 Effect on Inosine Content

As illustrated in Figure 12, the contents of nucleosides in dried samples varied under different MVD treatments. Compared to the natural drying group (0.27 mg/g), the mean values of inosine under the different drying temperatures were 0.258, 0.274, and 0.268 mg/g, respectively. The ANOVA result indicated no significant difference between the time gradients, suggesting that the different drying time conditions had a minimal effect on the inosine content of the dried PR. This can likely be attributed to the inherent thermal stability of inosine. However, a discernible decrease in inosine content was observed as the vacuum pressure increased (−60, −65, and −70 kPa). This reduction may be attributed to oxidative damage to the cell wall caused by the higher vacuum pressures, which could have led to the leakage of peroxidases from the cytoplasmic matrix. These enzymes likely catalyzed oxidation reactions involving inosine, thereby contributing to its decreased concentration (Zhang et al. 2022).

#### 3.8.2 Effect on Uracil Content

Uracil, an important medicinal component of PR, was found to be the least abundant nucleoside compared to other compounds such as guanosine, hypoxanthine, uridine, and inosine, a result consistent with previous studies (Wang et al. 2024). As drying temperature increased, the uracil content initially rose and then decreased. Specifically, the uracil content was 0.157 mg/g at 35°C, increased to 0.170 mg/g at 40°C, and then decreased to 0.158 mg/g at 45°C. This trend may be attributed to the fact that higher drying temperatures could reduce the activity of nucleoside phosphorylase slightly, leading to a subsequent decrease in uracil content. Additionally, varying vacuum pressures had significant effects on uracil content (*p* < 0.05), with the highest uracil concentration observed at a vacuum pressure of −60 kPa. This can be explained by the fact that higher vacuum pressures may alter the enzyme's protein structure, causing its binding pocket to become smaller, which subsequently reduced the specific activity of uracil phosphorylase (UPase).

#### 3.8.3 Effect on Adenosine Content

Adenosine, a key metabolite of nucleotides, plays a significant role in these processes. Figure 12 illustrates the effect of different PR drying conditions on adenosine content. In the naturally dried group, the adenosine content was 0.467 mg/g, which increased to 0.473 mg/g when the MVD temperature was set to 35°C. Furthermore, as the temperature was raised from 35°C to 45°C, the adenosine content showed a significant increase (*p* < 0.05). This could be due to elevated temperatures reducing the activity of adenosine deaminase, thereby limiting the degradation of extracellular adenosine (Chandel 2021). Under vacuum conditions, the changes in adenosine content followed a similar trend. This can be explained by the fact that higher vacuum levels may induce structural alterations in enzyme proteins, leading to a reduction in catalytic efficiency.

#### 3.8.4 Effect on Uridine Content

Figure 12 presents the values of the total uridine content of dried PR under different drying conditions. Drying treatment could gradually reduce the uridine content of PR. As the drying temperature increased from 35°C to 45°C, the uridine content of the dried samples increased from 1.636 mg/g to 1.886 mg/g (*p* < 0.05). This increase might be attributed to the short drying time at higher temperatures. Since uridine has good thermal stability, the higher drying temperature likely did not lead to thermal degradation of the uridine. On the contrary, with vacuum degree increased from −60 to −70 kPa, the uridine content of PR decreased from 2.090 to 1.547 mg/g. Generally, a lower vacuum degree corresponds to higher internal pressure. This decrease in uridine content could be due to stronger interactions between uridine and other chemical constituents under higher vacuum conditions, which might result in the reduction of uridine content.

#### 3.8.5 Effect on Guanosine Content

The effect of different PR drying conditions on the guanosine content is shown in Figure 12, where the guanosine content of the MVD dried samples decreased significantly after PR drying compared to the natural drying groups. As the drying temperature increased from 35°C to 45°C, the guanosine content first decreased from 2.183 mg/g to 1.785 mg/g, then rose to 2.038 mg/g. Similarly, as the vacuum degree increased from −60 kPa to −70 kPa, the guanosine content dropped from 2.149 mg/g to 1.785 mg/g, before increasing again to 2.030 mg/g. These changes are likely attributed to guanosine degradation via purine catabolism or the conversion of key intermediates under different drying conditions.

#### 3.8.6 Effect on Hypoxanthine Content

Hypoxanthine contents decreased significantly compared with the control groups such as natural drying and hot drying. The contents of hypoxanthine increased from 2.052 mg/g to 2.558 mg/g in different temperatures from 35°C to 45°C. This may be due to the increase of purine nucleosidase activity at high temperatures. Conversely, when the vacuum degree increased from −60 kPa to −70 kPa, the contents of hypoxanthine decreased gradually. This may be due to the disruption of the integrity of the matrix and modification of the structure of cell membranes and cell walls, facilitating the release of purine biosynthesis enzyme from tissues, thus leading to the decrease in content.

## 4 Conclusions

In this study, the impact of key drying parameters specifically drying temperature, vacuum degree, drying characteristics, physicochemical properties, and microstructural changes on the microwave vacuum drying (MVD) process of Pinelliae Rhizoma (PR) was thoroughly evaluated. This innovative drying method combined microwave and vacuum to overcome the problem of single‐stage drying. Different levels of vacuum degrees (−60, 65, and 70 kPa), temperature (35°C, 40°C, and 45°C) were applied to the Pinelliae Rhizoma (PR) by this hybrid drying technique.

The main findings of the study are as follows:

- The drying temperature increased from 35°C to 45°C, resulting in shorter drying times.
- The higher vacuum degree (−70 kPa) could lead to prolonging the drying time.
- The highest rehydration ratio measured was 2.22%, recorded at 40°C and −65 kPa, suggesting that the dried Pinelliae Rhizoma (PR) has a better rehydration capability.
- There were no significant differences in Δ*E*, suggesting that the combined effects of temperature, vacuum, pigment stability, and water migration resulted in similar color changes across different drying conditions.
- MVD at 40°C and −60 kPa resulted in a more compact surface microstructure, with denser internal cell structures in PR.
- Of the three drying models tested, the Midilli model exhibited the best predictive performance, corroborating the theoretical understanding of PR's drying characteristics.
- The Artificial Neural Network (ANN) model provided accurate predictions that aligned well with the testing data sets, offering valuable insights into understanding and controlling the factors affecting the drying process.
- According to the Principal component analysis and Pearson correlation analysis, MVD (40°C, −60 kPa) has a positive effect on the concentration of most nucleosides, with the exception of adenosine.

### 4.1 The Limitations of the Proposed Study

Although microwave drying offers numerous advantages compared to conventional drying methods, the drying conditions must be carefully controlled to mitigate potential disadvantages, such as the surface hardening and the loss of some active ingredients in Pinelliae Rhizoma.

### 4.2 Future Studies

Future optimization studies should focus on the application of hybrid systems to enhance drying quality and efficiency, possibly utilizing artificial intelligence technology to develop a more intelligent and easy‐to‐control microwave dryer to improve product quality and operational efficiency of dried Pinelliae Rhizoma (El‐Mesery, Qenawy, Li, et al. 2024).

**Nomenclature**
PR: Pinelliae Rhizoma
MVD: Microwave Vacuum Drying
a*: Redness/greenness coordinate in CIELAB color space
b*: Yellowness/blueness coordinate in CIELAB color space
L*: Lightness/darkness coordinate in CIELAB color space
Δ*E*
: Total color difference
BI: Browning Index
ANN: Artificial Neural Network
tansig: Hyperbolic tangent activation
purelin: linear transfer function
MSE: Mean Squared Error
*R* ^2^
: Coefficient of determination
*t*
: Drying time (min)
G: Absolute dry mass of Pinelliae Rhizoma (g)
DR: Drying Rate
*M₀*
: Initial moisture content (dry basis)
*Mₜ*
: Moisture content at time *t* (dry basis)
*M*_*R*_
: Moisture Ratio: *M*_*R*_ = *M*_*t*_/*M*_0_ *M*_*R*_ = *M*_*t*_/*M*_0_
*T*
: Drying temperature (°C)
*Wₜ*
: Weight of PR at time *t* (g)
HPLC: High‐Performance Liquid Chromatography
PCA: Principal Component Analysis
Rehydration Ratio: The ability to absorb water and restore its original freshness after drying
Maillard Reaction: Chemical reaction between amino acids and reducing sugars causing browning
Case Hardening: The phenomenon that the surface dries faster than the interior forms a hard layer that hinders the diffusion of moisture

## Author Contributions

**Pan Wang:** conceptualization (equal), formal analysis (equal), methodology (equal), software (equal), writing – original draft (equal). **Xiaopeng Huang:** supervision (equal), validation (equal), writing – review and editing (equal). **Guojun Ma:** visualization (equal). **Shidong Zhang:** visualization (equal). **Siqi Wang:** data curation (equal). **Ming Wang:** resources (equal). **Zewen Zhu:** visualization (equal). **Yanrui Xu:** investigation (equal). **ZePeng Zang:** investigation (equal). **Xu Liu:** investigation (equal).

## Ethics Statement

The authors have nothing to report.

## Consent

The authors have nothing to report.

## Conflicts of Interest

The authors declare no conflicts of interest.

## Data Availability

Data is contained within the article.
